# Supplementary material for: Continuous genomic diversification of long polynucleotide fragments drives the emergence of new SARS-CoV-2 variants of concern
Source: PNAS Nexus. 2022 Mar 10;1(1):pgac018. doi: 10.1093/pnasnexus/pgac018 (PMC9802374; doi:10.1093/pnasnexus/pgac018)
Supplement: pgac018_Supplemental_Files [file pgac018_supplemental_files.zip › PNASNEXUS-PNASNEXUS-2021-00270-s01.pdf]

Supplementary Material for:

Continuous genomic diversification of long polynucleotide fragments drives the emergence of new SARS-CoV-2 variants of concern

Karthik Murugadoss[1+], Michiel J.M. Niesen[1+], Bharathwaj Raghunathan[2], Patrick J. Lenehan[1], Pritha Ghosh[3], Tyler Feener[2], Praveen Anand[3], Safak Simsek[1], Rohit Suratekar[3], Travis K. Hughes[1], Venky Soundararajan[1,2,3]*

[1] nference, Cambridge, Massachusetts 02139, USA
[2] nference, Toronto, ON M5V 1M1, Canada
[3] nference Labs, Bengaluru, Karnataka 560017, India
+ Joint first authors

*Correspondence to: Venky Soundararajan (venky@nference.net)

Index

- **Figure S1:** Evaluating polynucleotide distinctiveness using different n-mer sizes.
- **Figure S2:** Map of SARS-CoV-2 VOC prevalence by geographic region.
- **Figure S3:** Polynucleotide distinctiveness analysis for various alternate sequence sets.
- **Figure S4:** 9-mer distinctiveness metric, considering the Delta Plus lineages separately and at an earlier time-point.
- **Figure S5:** Schematic of the methodology used in the n-mer distinctiveness analysis.
- **Figure S6:** Illustration of the geographical imbalance in the collection of SARS-CoV-2 sequence data.

- **Table S1:** Mapping of PANGO lineages to World Health Organization (WHO) nomenclature variant names used throughout the analysis.
- **Table S2:** Cohen's D values comparing the distributions of distinct nucleotide 9-mer counts in each VOC versus the original SARS-CoV-2 strain for various n-mer lengths.

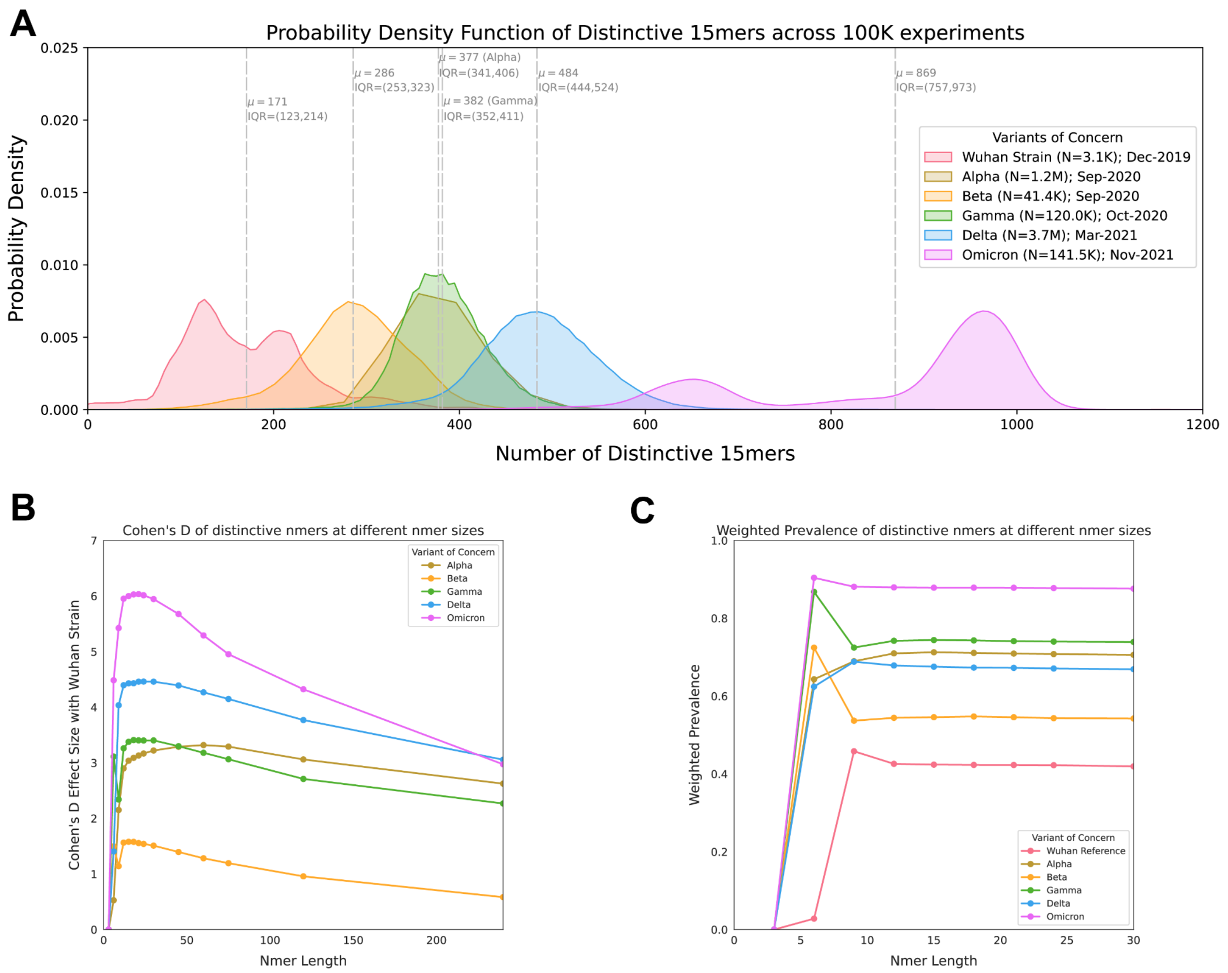

Figure S1: Evaluating polynucleotide distinctiveness using different n-mer sizes. (A) Density plots showing 15-mer sequence distinctiveness for VOCs, as measured by the number of distinct 15-mer polynucleotide sequences. **(B)** Cohen's D of the distinctive n-mer distributions of Alpha, Beta, Gamma, Delta, and Omicron variants against the original strain for various n-mer lengths (n = 3, 6, 9, 12, 15, 18, 21, 24, 30, 45, 60, 75, 120, and 240). **(C)** Intra-lineage prevalence of the distinctive n-mers found for the Alpha, Beta, Gamma, Delta, and Omicron variants for various n-mer lengths (n = 3, 6, 9, 12, 15, 18, 21, 24, 30). Prevalence is a weighted average over all distinctive n-mers for a variant, with weighting by the number of experiments in which a given n-mer was distinct to that variant.

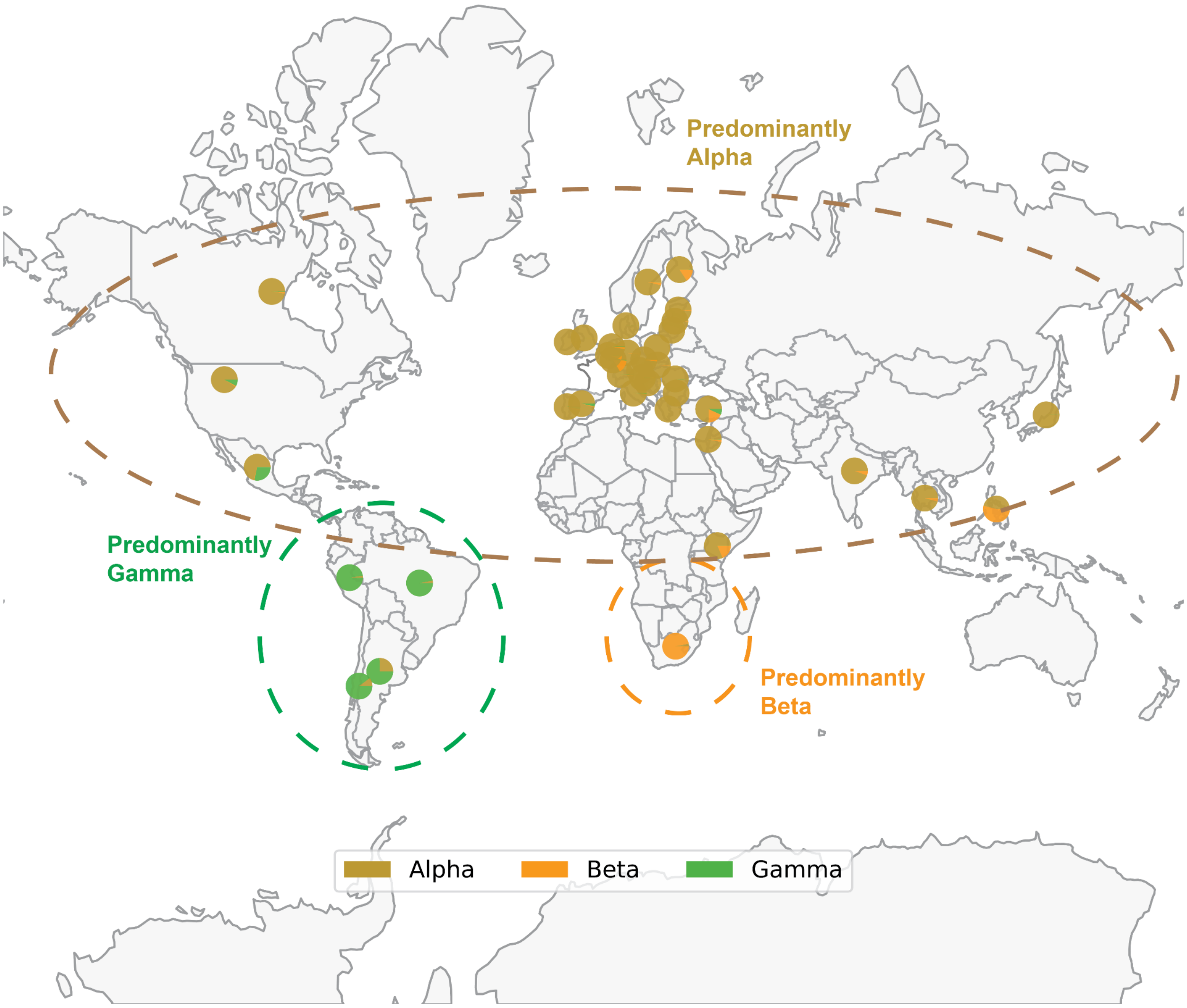

Figure S2. Map of SARS-CoV-2 VOC prevalence by geographic region. Geographical distribution of Alpha (B.1.1.7), Beta (B.1.351) and Gamma (P.1) variants based on sequences deposited in GISAID through December 14, 2021. Each pie chart shows the proportion of Alpha, Beta or Gamma sequences deposited in the country. Note that the denominator is the number of sequences labeled as any of these three variants, rather than the total number of sequences deposited in that country. Thus, each pie chart answers the following question: “Of all genomes deposited in a given country which were assigned as Alpha, Beta, or Gamma, what proportion of genomes was assigned to each of these three lineages?” The prevalence of Delta and Omicron are not shown to better highlight the geographical distribution of Alpha, Beta, and Gamma; however, Delta and Omicron are currently or have previously been highly prevalent in the regions shown. Only countries where at least 1000 sequences are deposited are shown. The variants depicted, which circulated at approximately the same time, generally became prominent in geographically distinct regions.

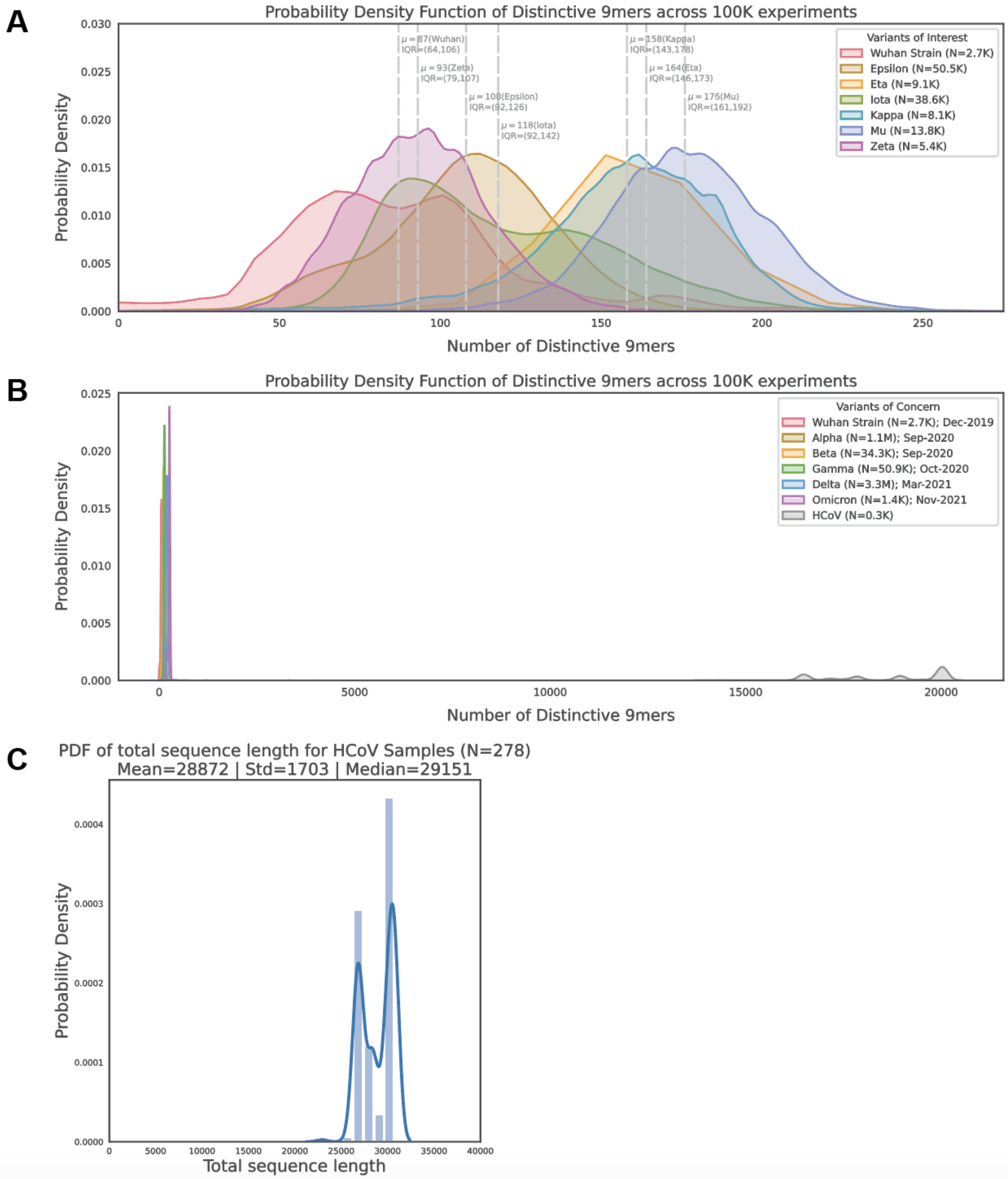

Figure S3. Polynucleotide distinctiveness analysis for various alternate sequence sets. (A) Distribution of the number of distinctive 9-mers for SARS-CoV-2 variants of interest. **(B)** Distribution of the number of distinctive 9-mers for SARS-CoV-2 variants of concern and human-infecting seasonal coronavirus (HCoV) genomes, showing that other virus species with similarly sized genomes are significantly more distinctive from the original SARS-CoV-2 strain than any of the SARS-CoV-2 VOCs considered. **(C)** Distribution of the genome length, in nucleotides, for the HCoV sequences used in panel (C). The genome length of SARS-CoV-2 is approximately 30 kilobases. Abbreviations: HCoV - human seasonal coronavirus.

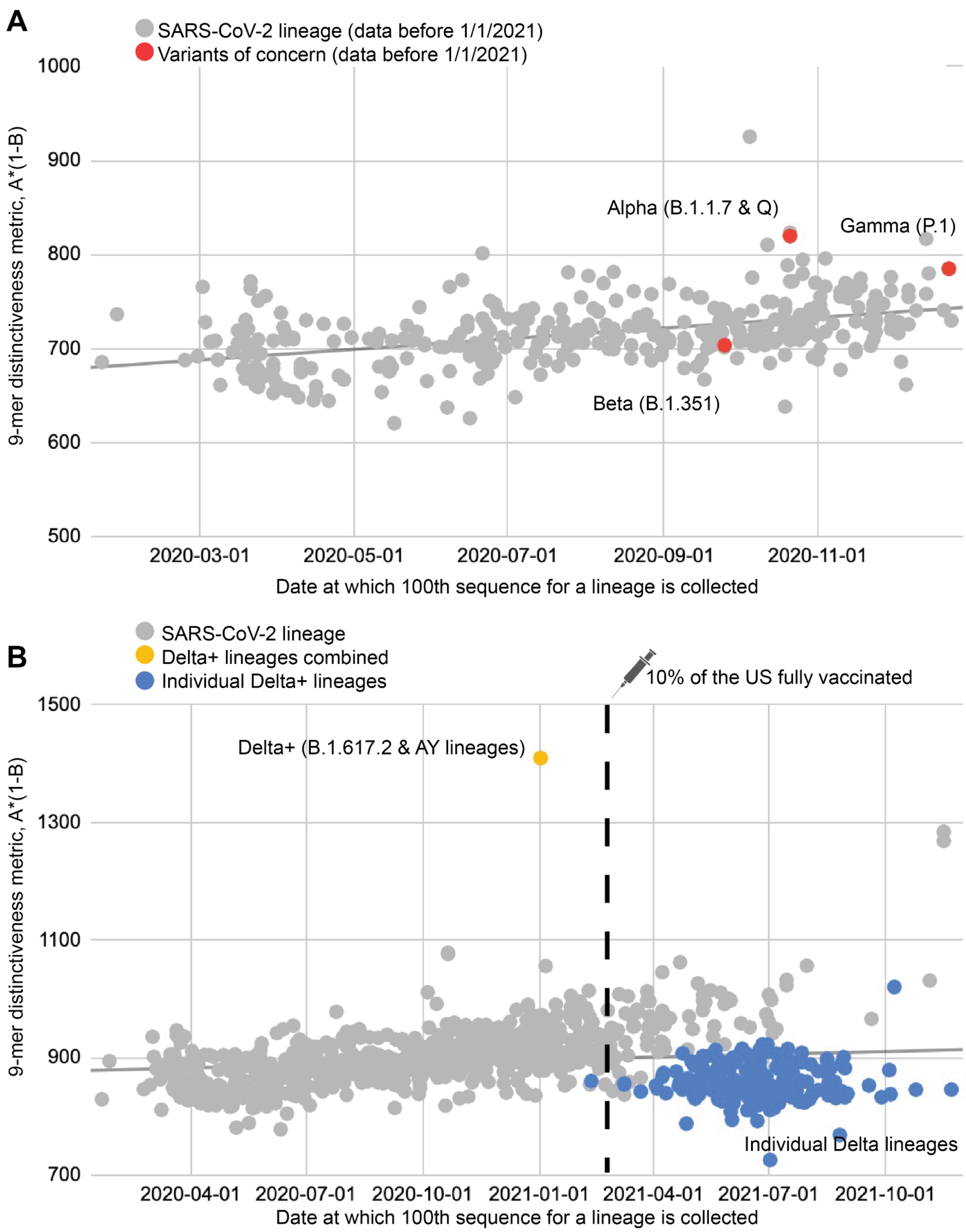

Figure S4. 9-mer distinctiveness metric, considering the Delta Plus lineages separately and at an earlier time-point. (A) Here we adapt Figure 7 from the main text of this manuscript, but only considering sequences collected prior to January 2021. This indicates that the Alpha variant was highly distinctive as assessed by this metric around its time of emergence. **(B)** Here we adapt Figure 7 from the main text of this manuscript, but with Delta Plus lineages also considered separately (blue dots).

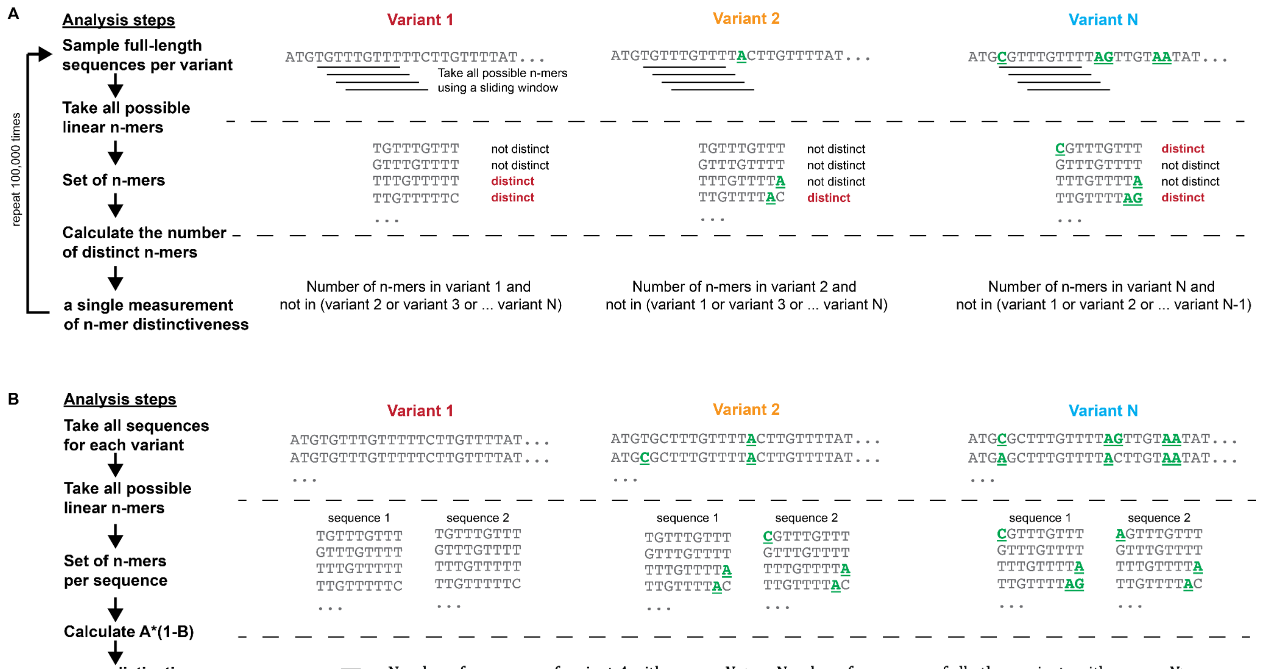

$$\sum_{N\in \text{n-mers}} \frac{\text{Number of sequences of variant } A \text{ with n-mer, } N}{\text{Number of sequences of variant } A}\left(1 - \frac{\text{Number of sequences of all other variants with n-mer, } N}{\text{Number of sequences of all other variants}}\right)$$

Figure S5. Schematic of the methodology used in the n-mer distinctiveness analysis. (A) The methodology used in the calculation of the number of distinct n-mers distributions shown in the main text. For illustration, a small part of a single sequence per variant is shown in the schematic; the main text analysis uses samples of six full-length sequences per variant, per measurement. **(B)** The methodology used in the calculation of the per-lineage alternative n-mer distinctiveness metric A*(1-B) that incorporates intra-lineage conservation scores. For illustration, a small part of two sequences per variant is shown; the main text analysis uses all available sequences that do not contain non-ATCG characters for each variant.

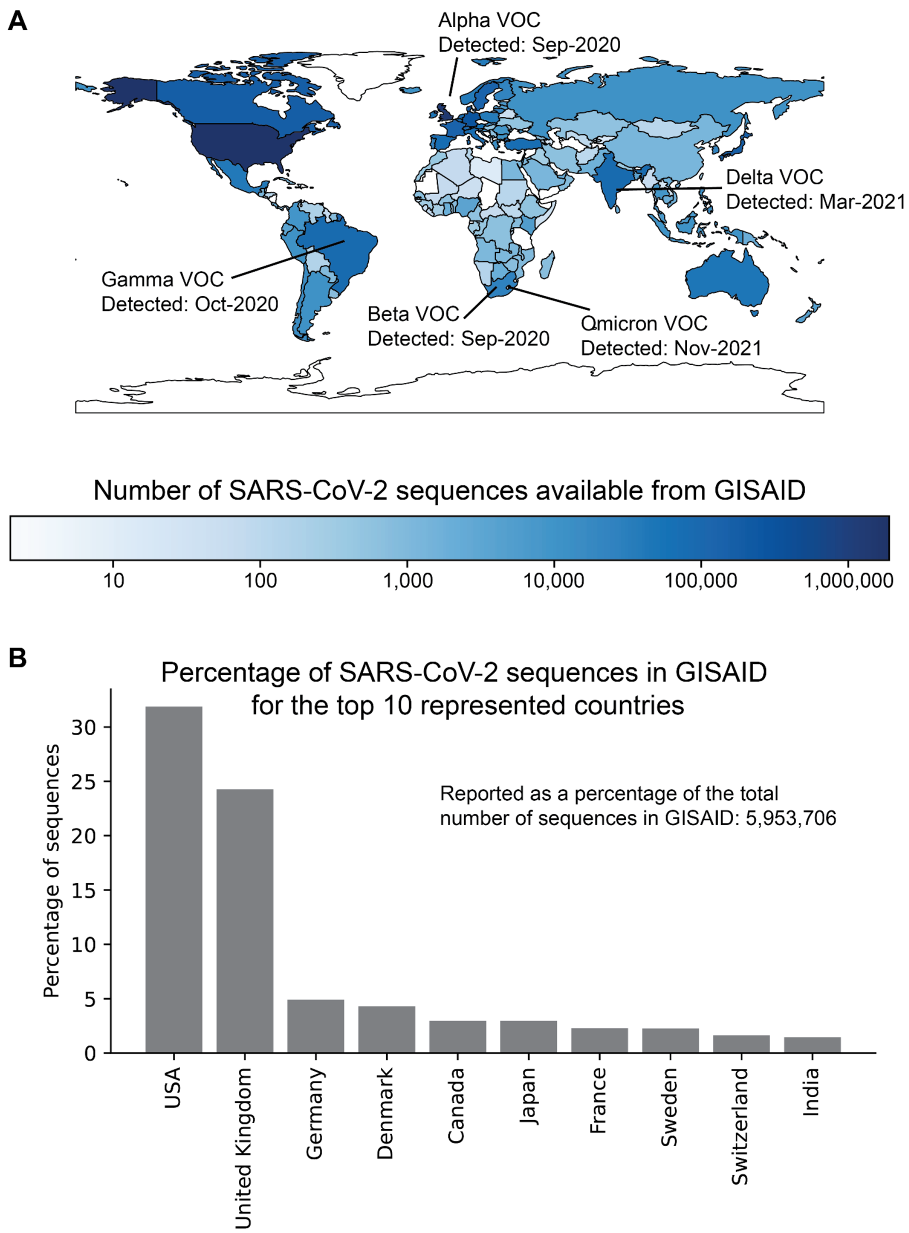

Figure S6: Illustration of the geographical imbalance in the collection of SARS-CoV-2 sequence data. (A) World map with each country or territory colored, using a logarithmic scale, according to the number of SARS-CoV-2 sequences available through the GISAID initiative that were collected in that region. **(B)** The percentage of sequences in GISAID that were collected in the top 10 most represented countries.

Table S1. Mapping of PANGO lineages to World Health Organization (WHO) nomenclature variant names used throughout the analysis. Source: https://www.cdc.gov/coronavirus/2019-ncov/variants/variant-classifications.html

Variant Name	PANGO Lineage (* indicates all descendant lineages)
Original Strain	A
Alpha	B.1.1.7, Q*
Beta	B.1.351*
Gamma	P.1*
Delta	B.1.617.2, AY*
Omicron	B.1.1.529, BA*
Epsilon	B.1.427, B.1.429
Eta	B.1.525
Iota	B.1.526
Kappa	B.1.617.1
Mu	B.1.621, B.1.621.1
Zeta	P.2

Table S2. Cohen's D values comparing the distributions of distinctive nucleotide 9-mer counts in each VOC versus the original SARS-CoV-2 strain for various n-mer lengths. The highest Cohen's D value for each variant is highlighted.

n-mer length	Alpha	Beta	Gamma	Delta	Omicron
3	0	0	0	0	0
6	0.53	1.5	3.11	1.4	4.49
9	2.15	1.14	2.34	4.04	5.42
12	2.9	1.56	3.26	4.4	5.95
15	3.04	1.58	3.38	4.43	6
18	3.09	1.58	3.41	4.43	6.03
21	3.13	1.56	3.4	4.46	6.03
24	3.17	1.54	3.4	4.46	6.01
30	3.22	1.51	3.4	4.46	5.95
45	3.29	1.4	3.3	4.39	5.68
60	3.32	1.28	3.18	4.27	5.29
75	3.29	1.19	3.06	4.15	4.95
120	3.06	0.96	2.71	3.77	4.32
240	2.63	0.58	2.27	3.06	2.97
